# A Profile of Cases of Gestational Trophoblastic Neoplasia at a Large Tertiary Centre in Dubai

**DOI:** 10.5402/2011/453190

**Published:** 2011-07-26

**Authors:** Tasneem H. Rangwala, Faiza Badawi

**Affiliations:** Department of Obstetrics and Gynaecology, Al Wasl Hospital, P.O. Box 9115, Dubai, United Arab Emirates

## Abstract

*Objectives*. To study (1) the prevalence of different types of gestational trophoblastic neoplasia (GTN) in the local and nonlocal population of women at Al Wasl Hospital, a tertiary level referral centre for northern Emirates, (2) the safety of cervical preparation before uterine evacuation, (3) the role of repeat uterine evacuation in curing these cases, and (4) the percentage of cases ultimately requiring chemotherapy. *Material and Methods*. Retrospective analysis of case records of 35 women with diagnosis of gestational trophoblastic neoplasia were managed in the Department of Obstetrics and Gynecology at Al Wasl Hospital, over a 2-year period between January 2007 to December 2008. *Results*. 35 cases of gestational trophoblastic neoplasia were seen in a 2-year period (January 2007 to December 2008) at Al Wasl Hospital, with 7000 deliveries per year, prevalence being 1 in 400 live births. 60% cases were local Arabs. Histopathology revealed complete mole in 13 cases, partial mole in 17 cases, hydropic degeneration of villi in 4 cases, and no identifiable tissue in 1 case. No cases of choriocarcinoma or placental site trophoblastic tumour were seen during the study period. 34% cases received cervical preparation with prostaglandins prior to surgical curettage. Complications were minor. 62% were cured by primary suction curettage, 12% after second (repeat) uterine evacuation, and 25% needed single drug chemotherapy. 8% cases defaulted after primary evacuation and were lost to followup. *Conclusions*. Prevalence of GTN in the local Arab population is similar to other Asian populations. The majority of cases are cured by simple suction uterine curettage. Cervical preparation with prostaglandins should be done in selected cases to avoid perforation during evacuation. Second (repeat) uterine evacuation can be curative in some cases with strict selection criteria and avoid the need for chemotherapy. Regional registry of cases is needed to estimate the true incidence of this disease.

## 1. Introduction

Gestational trophoblastic neoplasia is excessive and inappropriate proliferation of trophoblast after the pregnancy has ended. It includes a spectrum of disease from the benign hydatidiform mole (complete or partial mole), to the malignant gestational trophoblastic tumour (invasive mole, choriocarcinoma, and placental site trophoblastic tumour). It is a rare but important pregnancy-related disorder with an incidence of 1 in 400 in Asia and Latin America. The majority of cases can be cured by simple surgical intervention. Those cases requiring chemotherapy are generally cured with very low toxicity regimen. Unlike other gynaecological malignancies, fertility can be preserved and normal pregnancy outcome anticipated [1]. The curability of this condition is a milestone of success in the history of modern medicine. However, the disease can recur, and referral to a specialist centre is required to ensure proper monitoring and followup of cases. We present a retrospective study of cases of GTN over a 2-year period that were managed at our hospital, which is a tertiary level teaching hospital.

## 2. Material and Methods

Gynaecological case records of 35 cases who were admitted with the diagnosis of GTN during Jan. 2007 to Dec. 2008 were analysed and data recorded in a data collection tool designed for the purpose of this study (Table 1). Data were stored in Excel format and analysed, and descriptive statistics were used. Cases were managed according to institutional protocol based on RCOG (Royal College of Obstetricians and Gynaecologists) guidelines [2]. All cases admitted from Accident & Emergency or Early Pregnancy Assessment Unit at Al Wasl Hospital with suspected diagnosis of molar pregnancy had detailed pelvic ultrasound, chest X-ray, and Laboratory investigations including full Blood count (FBC), Blood group and Rh typing, and baseline serum *β*-HCG. Suction curettage was done under general anaesthesia for all cases by experienced staff. One case presenting at 24-week gestation with partial molar changes on ultrasound had medical termination with Misoprostol (prostaglandin E1). Prior cervical preparation with prostaglandins (PG E1 OR E2) was done in selected cases with tightly closed cervical os and history of previous uterine surgery. Oxytocin injection was given after evacuation if there was excessive haemorrhage. Evacuated material was sent for histopathology by experienced pathologists. These women were followedup in Gynecology Outpatients Clinic, by weekly serum *β*-HCG levels till 3 values were negative, then monthly for 6 months, and 3 months for 1 year. If *β*-HCG levels showed rise or plateau during followup, cases were readmitted for further ultrasound and managed as persistent trophoblastic disease. If ultrasound showed significant retained tissue and *β*-HCG less than 1500 IU/L, re-evacuation under general anaesthesia was done by senior level staff. After histopathology report, followup was continued with *β*-HCG, and the case was referred to Medical Oncology Department for chemotherapy evaluation. Contraception was advised for 1 year till followup completed.

## 3. Results

35 cases of GTN were seen in 2 years (January 2007 to December 2008) with 7000 deliveries per year, giving a prevalence of 1 in 400 live births. On average, 1 to 3 cases were encountered in a month. The Age of women in the study ranged from 17 to 49 years. Maximum number of cases were seen in 21 to 30 years age group. Parity ranged from 0 to 10, and the majority were seen in the parity group 1 to 4. 

**Figure 1:**
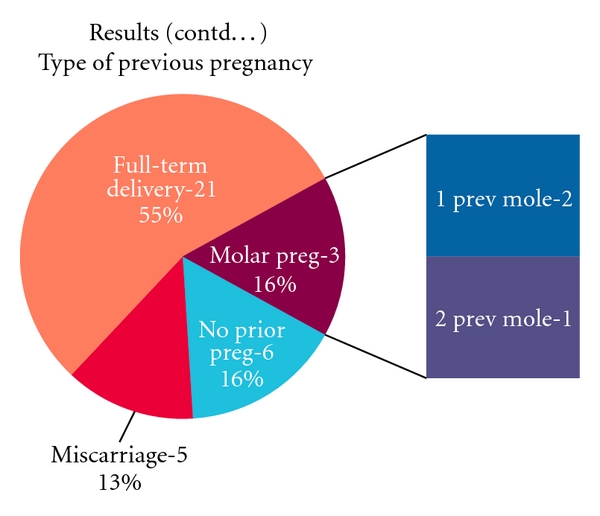
Past obstetric history.

 21 women (60%) were local Arab nationals, while 14 (40%) were expatriates. Amongst the expatriates, 7% were European and remaining were Asians of different nationalities. The type of previous pregnancy in these cases is shown in Figure 1. 51% of cases had no symptoms on presentation and were diagnosed on early pregnancy scan. The remaining presented with abdominal pain (6 of 35—17%), bleeding per vaginum (15 of 35—29%), and hyperemesis (1 of 35—3%). Also 54% had no presenting signs. The remaining showed Large for dates uterus (12 of 35—34%), Theca lutein ovarian cysts (3 of 35—9%), and pre-eclampsia (1 of 35–3%).

**Figure 2:**
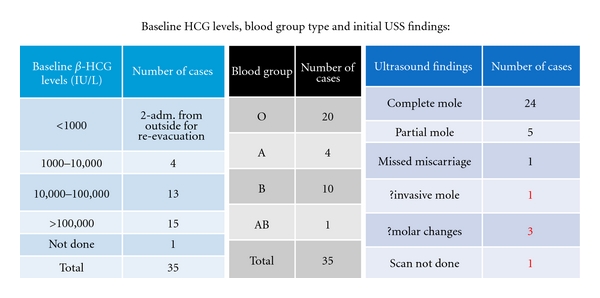
Initial investigations.

 The baseline *β*-HCG levels, ultrasound findings, and blood group are shown in Figure 2. *β*-HCG level of more than 1 lac units per litre was seen in the majority of cases, and predominating blood group was “O” followed by “B”. Ultrasound showed clear picture of complete mole in 24 cases and partial mole in 5 cases. 

**Figure 3:**
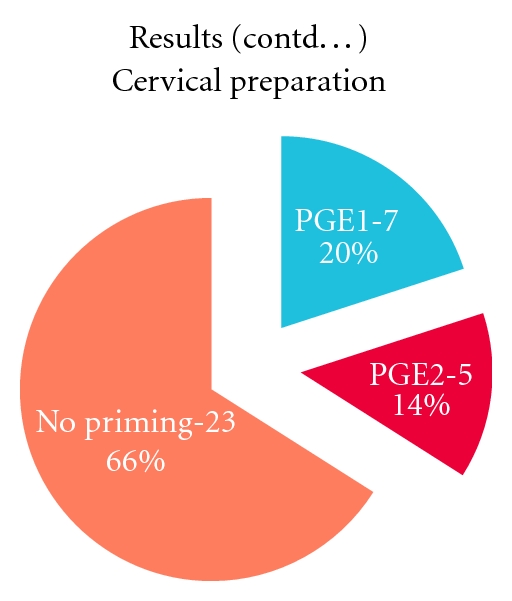
Type of cervical preparation.

 The type of cervical preparation used is shown in Figure 3. 34% of cases had received prostaglandin preparation. Suction curettage was done in 34 cases (97%) and medical termination using PGE1 (misoprostol) in 1 case (3%) which presented at 24 weeks with severe pre-eclampsia and partial mole. 14 of 34 cases received oxytocin infusion after evacuation to control hemorrhage. Intraoperative and immediate postoperative complications were minimal. Cervical laceration was seen in 1 (3%), fever in 1 (3%), hemorrhage in 2 (6%), and none in 31 cases (88%). 

**Figure 4:**
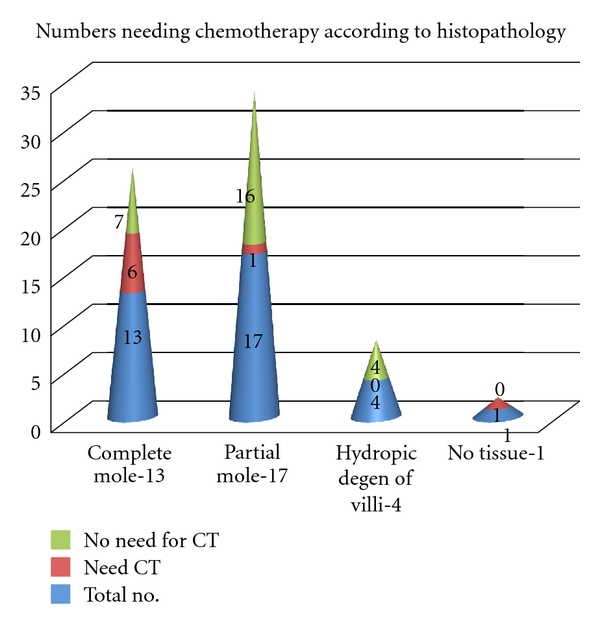
Histopathological subtypes

 The histopathology type and number of each type needing chemotherapy are shown in Figure 4. 13 were complete moles (6 needed chemotherapy), 17 were partial moles (1 needed chemotherapy), and 4 were hydropic degeneration of villi. No cases of invasive mole, choriocarcinoma, or placental site trophoblastic tumour were seen in the study group. Hence, 8 of 35 cases (22.8%) underwent chemotherapy. All these cases received single-agent methotrexate regimen with folinic acid as their FIGO risk score was low (0 to 6). Cervical priming with prostaglandin was done in 1 out of these 8 cases which required chemotherapy. Second evacuation was attempted in 7 out of these 8 cases. 

**Figure 5:**
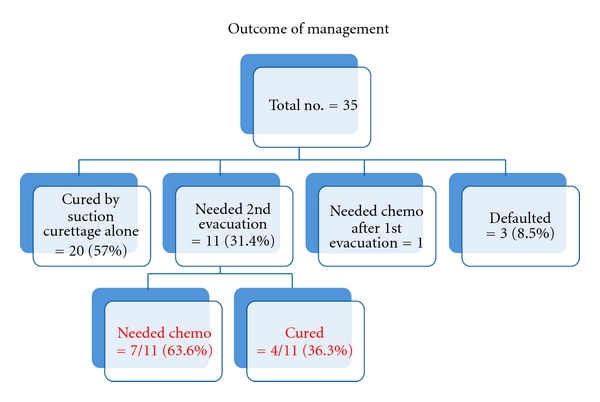
Management outcome.

 30 cases (85.7%) had regular *β*-HCG followup, irregular in 4 cases, and none in 1 case. 19 cases were followed up for one year, 13 cases for less than 6 months, and three cases defaulted. Probably some went to their country of origin for followup.  Figure 5 shows the outcome of management in our institution. As seen in the figure, 20 cases (68%) were cured by first suction curettage and four (12%) by second curettage. 8 cases (25%) needed chemotherapy.

## 4. Discussion

GTN is a rare disease with varied presentation, and clinicians are still faced with many challenges in management. It includes both benign and malignant forms. The benign forms have excellent prognosis with 100% cure rate after simple surgical evacuation, and 5 to 8% need chemotherapy. Early diagnosis by ultrasound, availability of sensitive *β*-HCG assays, and introduction of effective chemotherapy regimens have made this once fatal malignancy curable. When presenting at early gestations, complete mole can be differentiated from partial mole by histopathology of evacuated material and ploidy test. Immunostain with P^57^KIP2 confirms complete mole [2]. 

 The incidence of GTN varies from 1 in 1000 in the west to 1 in 100 in Philippines. In our study, the prevalence was 1 in 400 live births. The actual incidence could not be calculated as there is no national registry for this tumour. GTN is common at extremes of age, less than 15 years and more than 45 years [2]. The increased risk in these age groups is mainly for complete molar pregnancy as the incidence of partial molar pregnancy changes less across the age groups [2]. In our study, the majority was seen in 21 to 30 years age group. It is known that molar pregnancy can recur [2], the recurrence being 1 in 60 after 1 molar pregnancy, 1 in 10 after 2 molar pregnancies, and 1 in 2 after 3 molar pregnancies. In our study, 2 out of 35 cases had history of one previous molar pregnancy, and 1 case had history of 2 previous molar pregnancies. The recurrence is usually of the same histopathological type [3].

 These cases usually present with bleeding per vaginum, early pregnancy failure, hyperemesis, or excessive uterine enlargement. Pre-eclampsia and hyperthyroidism are rare presentations [4]. In our study, 50% cases had no signs or symptoms at presentation and were diagnosed by early pregnancy scan. Nowadays, early diagnosis is possible by ultrasound and Doppler [4]. In our study, 33 out of 35 cases were diagnosed by pre-evacuation scan. Complete mole appears as anembryonic pregnancy and partial mole as cystic spaces in placenta or transverse to anteroposterior sac diameter >1.5. 

 When cervical preparation is done using prostaglandin, there is a theoretical risk of embolisation of trophoblastic cells, and prolonged preparation should be avoided [4, 5]. A case-controlled study of 219 patients [5] showed no evidence that cervical ripening prior to evacuation was linked to higher risk for needing chemotherapy, but there was a link with *increasing* uterine size and subsequent need for chemotherapy [5]. In our study, 1 out of the 8 cases who received cervical preparation needed chemotherapy. We used prostaglandin preparation for selected cases with tightly closed os and history of previous uterine surgery.

 Complications associated with GTN include hemorrhage, tumour embolisation, uterine perforation, and sepsis. These were minimal in our study. Uterine perforation during evacuation can be minimized if procedure is done by experienced personnel.

 15% of complete moles and 0.5% of partial moles need chemotherapy [2]. In our study, 6 out of 13 cases of complete moles and 1 out of 17 cases of partial moles needed chemotherapy. The true incidence cannot be calculated as all molar pregnancies are not registered at one centre. The risk of requiring chemotherapy is more when molar pregnancy presents at an earlier gestation [6].

 Repeat or second uterine evacuation has a role in selected cases of persistent gestational trophoblastic disease where *β*-HCG is less than 1500 IU/litre, and ultrasound shows significant retained molar tissue. Chemotherapy can be avoided in these cases. Chemotherapy is more likely if there is histological evidence of persistent gestational trophoblastic disease, and *β*-HCG is more than 1500 IU/litre at the time of repeat uterine evacuation. Third evacuation is no longer recommended [7]. In our study, 4 out of 11 cases who underwent second evacuation were cured, while 7 cases ultimately needed chemotherapy. This indicates the need for strict selection criteria.

 Followup of patients can be improved by adequate counselling at the time of diagnosis. Nowadays, shortened duration of followup is advised for 6 months from the date of evacuation if *β*-HCG becomes normal within 8 weeks of evacuation. If *β*-HCG does not revert to normal within 8 weeks of evacuation, then followup is advised for 6 months from the date of normalisation of the *β*-HCG. This reduces anxiety for women and is cost effective [4, 8, 9].

## 5. Conclusions

 The prevalence of GTN among the Arab population appears to be similar to other Asians, and there is need for establishing a national registry to obtain the true incidence of the disease. Appropriate diagnosis and treatment leads to near 100% cure. Majority of cases are cured by simple surgical intervention (suction curettage). Cervical preparation is safe in selected cases. Second evacuation should be reserved for cases with strict selection criteria. Followup can be improved by adequate counselling.

## 6. Learning Points/Recommendations

1. There should be a regional/national registry for GTN where all these cases are registered and receive appropriate followup.
2. All cases should have a baseline *β*-HCG level and pelvic ultrasound before any intervention.
3. Caution during uterine evacuation can avoid serious complications.
4. Cervical preparation with prostaglandin should be done in selected cases, and prolonged preparation should be avoided.
5. Repeat evacuation in selected cases avoids need for chemotherapy.
6. All cases of persistent GTN should have FIGO risk scoring and receive chemotherapy in specialised centres.
7. Followup is ideal for one year, but cases should be counselled for contraception and refrain from pregnancy for at least 6 months.
8. Six weeks after the end of any subsequent pregnancy, serum *β*-HCG sample should be evaluated, and placenta should be sent for histopathology after any delivery.

## Figures and Tables

**Table 1a:** (a) Page-1

HC no:	Age	Parity	Symptoms	Signs	Baseline	USG findings	Prev. preg	Cx priming	Suction evacuation
	(Yrs.)		Bleeding/vesicles	LFD uterus/ov cysts	B-HCG	Mole/missed		Yes/No	Yes/No

									

									

									

									

**Table 1b:** (b) Page-2

Oxytocin	Complication	B-HCG F/U	2nd Evac	Outcome	F/U duration (months)	Histopath	Blood grp
Yes/No		Reg/Irreg	Yes/No	Cured/need CT			

							

							

							

							

## References

[1] Tse KY, Chan KKL, Tam KF, Ngan HYS (2009). Gestational trophoblastic disease. *Obstetrics, Gynecology and Reproductive Medicine*.

[2] savage P (2008). Molar pregnancy. *The obstetrician and Gynecologist*.

[3] Sebire NJ, Fisher RA, Foskett M, Rees H, Seckl MJ, Newlands ES (2003). Risk of recurrent hydatidiform mole and subsequent pregnancy outcome following complete or partial hydatidiform molar pregnancy. *British Journal of Obstetrics and Gynaecology*.

[4] Royal College of Obstetricians and gynaecologists The management of Gestational Trophoblastic disease.

[5] Flam F, Petterson F, Lundstrom V (1991). Medical induction prior to surgical evacuation of Hydatidiform mole. Is there a greater risk of persistent gestational trophoblastic disease?. *European Journal of Obstetrics and Gynecology and Reproductive Biology*.

[6] Stone M, Bagshawe KD (1979). An analysis of the influences of maternal age, gestational age, contraceptive method, and the mode of primary treatment of patients with hydatidiform moles on the incidence of subsequent chemotherapy. *British Journal of Obstetrics and Gynaecology*.

[7] Pezeshki M, Hancock BW, Silcocks P (2004). The role of repeat uterine evacuation in the management of persistent gestational trophoblastic disease. *Gynecologic Oncology*.

[8] Pisal N, Tidy J, Hancock B (2004). Gestational trophoblastic disease: is intensive follow up essential in all women?. *British Journal of Obstetrics and Gynaecology*.

[9] Sebire NJ, Foskett M, Short D (2007). Shortened duration of human chorionic gonadotrophin surveillance following complete or partial hydatidiform mole: evidence for revised protocol of a UK regional trophoblastic disease unit. *British Journal of Obstetrics and Gynaecology*.

